# The significance of targeting lysosomes in cancer immunotherapy

**DOI:** 10.3389/fimmu.2024.1308070

**Published:** 2024-02-02

**Authors:** Yanxin Xu, Bo Shao, Yafeng Zhang

**Affiliations:** ^1^ Department of Colorectal Surgery, The First Affiliated Hospital of Zhengzhou University, Henan, Zhengzhou, China; ^2^ Institute for Hospital Management of Henan Province, The First Affiliated Hospital of Zhengzhou University, Zhengzhou, China

**Keywords:** lysosomes, tumor immunity, immunotherapy, tumor microenvironment, lysosomal autophagy

## Abstract

Lysosomes are intracellular digestive organelles that participate in various physiological and pathological processes, including the regulation of immune checkpoint molecules, immune cell function in the tumor microenvironment, antigen presentation, metabolism, and autophagy. Abnormalities or dysfunction of lysosomes are associated with the occurrence, development, and drug resistance of tumors. Lysosomes play a crucial role and have potential applications in tumor immunotherapy. Targeting lysosomes or harnessing their properties is an effective strategy for tumor immunotherapy. However, the mechanisms and approaches related to lysosomes in tumor immunotherapy are not fully understood at present, and further basic and clinical research is needed to provide better treatment options for cancer patients. This review focuses on the research progress related to lysosomes and tumor immunotherapy in these

## 1 Introduction

For an extended period, lysosomes have primarily been considered integral components of cellular degradation and recycling due to their inherent acidity and rich complement of hydrolytic enzymes, facilitating the breakdown of cellular constituents and extracellular molecules into their constituent components (1). However, with the deepening of research, it has become evident that lysosomes play a broader role in various cellular processes. This is mediated through multiple mechanisms, including the mammalian target of rapamycin complex 1 (mTOR1) (2, 3), Ca^2+^ ion efflux, transcription factor EB (TFEB) nuclear translocation (4), as well as the differential localization of lysosomal membrane proteins and associated proteins (5–7). Lysosomes are now recognized as crucial mediators of cellular material transport, encompassing processes such as autophagy, endocytosis, and phagocytosis, while also significantly contributing to proliferation, differentiation, metabolic regulation, secretion activities, and the quality control of protein aggregates and damaged organelles (1, 8, 9). They have emerged as multifaceted hubs for immune regulation, nutrient sensing, and information trafficking. Any alterations or functional impairments in lysosomes have the potential to disrupt the intrinsic equilibrium of cells and tissues, leading to or exacerbating human diseases, including cancer (10, 11).

The process of tumor initiation and progression is closely intertwined with the contribution of the tumor microenvironment (TME) (7, 12). The TME constitutes a complex biological system surrounding tumor cells, including non-tumor cells such as immune cells, fibroblasts, endothelial cells, extracellular matrix (ECM), blood vessels, lymphatic vessels, signaling molecules, and metabolites, among others (7, 12). Among these components, lysosomes play an indispensable role in regulating immune functions, antigen presentation, and immune evasion (13). Antigen-presenting cells (APCs) primarily rely on lysosomes to engulf exogenous antigens, process them into peptides, and transport them to the major histocompatibility complex class II (MHC-II) loading compartment (14). Alternatively, they can also utilize autophagosomes to target cytoplasmic proteins for lysosomal degradation, thereby activating CD4^+^ helper T cell responses and coordinating specific immune reactions (15). However, the major histocompatibility complex class I (MHC-I), when bound to the autophagy cargo receptor NBR1, undergoes autophagic/lysosomal co-localization degradation, impairing the immune surveillance function of CD8^+^ T cells (16). Additionally, lysosomes are involved in the intracellular trafficking, presentation, and degradation of co-stimulatory molecules and immune checkpoint proteins (17). For instance, the co-localization of CKLF-like marvel transmembrane domain-containing protein 6 (CMTM6) with programmed cell death ligand 1 (PD-L1) on the plasma membrane and recycling endosomes of cancer cells prevents PD-L1 lysosomal degradation, thereby inhibiting the activity of tumor-specific T cells (18). CD47, widely expressed on the surface of various cancer cells, interacts with the macrophage-expressed SIRPα, generating a “don’t eat me” signal (19). Lysosomal protein RAGA drives the endocytosis of CD47 and relocalizes it to lysosomes for degradation, thus restoring macrophage phagocytic signaling (20). Furthermore, lysosomes influence immune responses by modulating the lineage differentiation pathways of immune cells. After antigen stimulation, the mTOR signaling pathway becomes activated, guiding immune cells towards various functional lineage differentiations. Lack of mTOR in precursor cells, even when activated and maintaining IL-2 levels, fails to differentiate into Th1, Th2, or Th17 effector cells, instead favoring the differentiation into regulatory T cells (Tregs) (21). However, sustained activation of the mTOR signal may lead to T cell functional exhaustion, resulting in terminal differentiation and reduced proliferative potential (22), potentially weakening the anti-tumor response. Furthermore, evolutionarily conserved autophagy-related genes such as ATG5 are involved in the renewal, differentiation, and homeostasis maintenance of most immune cells (23). For instance, macrophages differentiated from peripheral blood monocytes, stimulated by granulocyte-macrophage colony-stimulating factor (GM-CSF), release BECL1 and undergo ATG5 cleavage, triggering autophagic signaling to promote the survival of differentiated macrophages (24). However, some studies have found that inhibiting autophagy can promote macrophage differentiation from myeloid progenitor cells (25). Additionally, efficient B cell development in the bone marrow and the maintenance of the number of peripheral B-1a B cells, independent of antigen influence, rely on the autophagy gene ATG5 (25). Activated B cells entering the germinal center require a transition from classical autophagy to non-canonical LC3-associated autophagy to differentiate into long-lived plasma cells and memory B cells (26). In summary, these findings suggest that lysosomes play a multifaceted role in shaping the immune response and its intensity regulation, contributing significantly to the modulation of the tumor immune microenvironment.

Tumor immunotherapy is a treatment approach that harnesses the body’s own immune system to recognize and eliminate cancer cells. It offers advantages such as high specificity, long-lasting effects, and minimal toxicity. It represents a new generation of cancer treatment methods that have rapidly developed following traditional treatments like surgery, radiation therapy, and chemotherapy (27, 28). Currently, immunotherapy methods applied in clinical practice primarily include immune checkpoint inhibitors (ICIs), adoptive cell therapy (ACT), and cancer vaccines, among others (29–31). In recent years, the pivotal role of lysosomes in supporting cancer cell survival and regulating the TME has garnered increasing attention. This has led to lysosomes being considered a promising therapeutic intervention target (32–34). Combining emerging technologies, various targeted lysosome-based combination immunotherapies have been developed. For instance, Tang et al. reported a pH-gated nano-adjuvant (PGN) that selectively targets lysosomes in tumor-associated macrophages (TAMs), mitigating lysosomal acidity and protease activity to polarize them towards an M1 phenotype, thereby enhancing antigen cross-presentation (35). Targeting lysosome degradation functions, on one hand, synergizes with ICIs. Lysosome-targeting chimeric molecules (LYTACs) have been designed to degrade cell membrane proteins (34), with one end binding to the target protein through antibodies or small molecules, and the other end being an oligosaccharide peptide, effectively targeting CI-M6PR through the endocytic/lysosomal pathway to successfully degrade epidermal growth factor receptor (EGFR) and PD-L1 (36). On the other hand, it can also synergize with tumor vaccines to improve vaccine specificity and enrichment. For instance, Deng et al. designed mRNA vaccines based on sialic acid-cholesterol derivatives, ensuring both dendritic cells (DCs) targeting and endosomal escape (37).

Targeting lysosome trafficking processes inhibits the endosomal sorting complex required for transport (ESCRT)-mediated repair of small membrane wounds in cancer cells, promoting the release of granular enzymes from tumor-infiltrating T cells (TILs) into the cytoplasmic solute (33). Targeting lysosome toll-like receptors (TLRs) activates innate immunity and modulates the responsiveness of TME to immunotherapy. For example, when the cancer vaccine NY-ESO-1 is combined with the TLR3 agonist poly-ICLC, the immune response against the cancer/testis antigen NY-ESO-1 is enhanced (38). Additionally, chloroquine, as the only autophagy inhibitor currently used in clinical cancer therapy, has been shown to significantly improve the therapeutic efficacy of combined ICIs in various cancers (16, 39, 40).

In summary, lysosome targeting, a novel cancer immunotherapy strategy, disrupts tumor-immune interactions in the TME through multiple mechanisms, thereby enhancing or synergizing with immunotherapy outcomes. Although the aforementioned is appealing and full of potential, it currently faces several challenges such as the need to overcome issues with selective drug delivery, safety, and resistance, as well as to explore further the precise underlying mechanisms.

## 2 Basic characteristics of lysosomes

Lysosomes, as the principal degradative component of eukaryotic cells, are responsible for the primary and ultimate degradation as well as recycling processes within the cell (1). In addition to this, lysosomes, in coordination with other organelles, also participate in various physiological processes such as cholesterol homeostasis, membrane repair, bone and tissue remodeling, pathogen defense, cell death, and cell signaling (4). These multifaceted functions render lysosomes a central and dynamic organelle, not merely confined to the terminal point of endocytic pathways (41). Lysosomes are encapsulated by a double-layered membrane, forming vesicles rich in specific soluble hydrolytic enzymes, integral membrane proteins, and lysosome-associated proteins. The lumen of lysosomes contains over 60 types of acidic hydrolytic enzymes, along with enzyme activators. They are responsible not only for intracellular degradation and proprotein processing but also play roles in antigen processing, degradation of the extracellular matrix, and initiation of apoptosis (5). The function of these hydrolytic enzymes is contingent upon the acidic pH (4.0-5.0) within the lumen, maintained by the vacuolar ATPase embedded in the limiting membrane (42). Furthermore, more than 30 known integral membrane proteins are highly glycosylated and serve functions such as protecting the lysosomal membrane from degradation, importing proteins from the cytoplasm, membrane fusion, and transporting degradation products to the cytoplasm, ion exchange, etc. Examples include lysosome-associated membrane proteins 1 and 2 (LAMP1/2), lysosome integral membrane protein 2 (LIMP2), and SNAREs (43, 44). Specific lysosome-related molecules are detailed in **Table 1**. Most importantly, the recruitment of the mechanistic target of mTOR1 to lysosomes is facilitated by RAG-GTPases, coupling amino acid supply with cell growth and autophagic signals (3). Triggered in a manner dependent or independent of mTOR1, TFEB undergoes nucleocytoplasmic shuttling, regulated by phosphorylation. This promotes the formation of autophagosomes, autolysosomes, and lysosome-dependent cell death, among other processes (45). Rab GTPases recruit and activate tethering factors for contact with other organelles, coupling lysosomes with motor complexes, such as dynein-dynactin and kinesin motors, which drive vesicle movement along microtubules (46).

**Table 1:** Lysosomal component functions.

Location	Protein property	Concrete molecules				
Lysosome components	Hydrolases	GBA, GAA, HEXB, NEU1, ACP5, GNS, PPT1, SGSH, NAGLU, TPP1				
	Membrane protein	LAMP-1/2	Lysosomal stability and integrity; Lysosomal exocytosis			
		LIMP-2	Biogenesis and maintenance of endosomes/lysosomes; Mannose 6-phosphate			
		v-ATPase	Acidic pH regulation of the lysosomal lumen (ATP6V0B, ATP6V0E1, ATP6V1H, ATP6V1C1, ATP6V1E1, ATP6V1G1, ATP6V1A)			
		Transport protein	Ca^2+^ Ion channel: TRPML1, TPC, P2X_4_			
			L-cystine amino acid/H+: Cystinosin			
			Cl^-^/H^+^ Ion channel: CIC7, OSTM1			
			Cholesterol/Fatty acid transport: NPC1 (LIMP-2, PTCH1)			
			Ialic acids, Aspartate, Glutamate transport: Sialin:			
			Arginine transport: CLN3			
		SNAREs	Late endosomes/ lysosomes		Syntaxin 7 (Q), Syntaxin 8 (Q), VTI1B (Q), VAMP7/VAMP8 (R)	small GTPases (ARL8, RAB7) tethering factors (HOPS, EPG5, PLEKHM1, ATG14L)
			Autophagosomes		Syntaxin 17 (Q), SNAP29 (Q), VAMP7/VAMP8 (R)	docking proteins (LC3, GABARAP) tethering factors (EPG5, ATG14L)
			Plasma membrane		Syntaxin4 (Q), SNAP23 (Q), VAMP7 (R)	PtdIns (4, 5) P2, synaptotagmin7 (SYT7)
	Transcription factors	TFEB, MITF, TFEC, TFE3				
	Signaling pathways	Rag-Regulator-v-ATPase→mTORC1 (mTOR, DEPTOR, MLST8, PRAS40, RAPTOR)→ULK1, TFEB, UVRAG, ATG14L				
Autophagosome biogenesis	Cargo recruitment	p62, TAX1BP1, NBR1				
	Elongation	ATG5				
	Conjugation	ATG4, ATG8				
	Membrane carrier	ATG9, ATG2				
	Maturity	VPS34, ATG14, BECN1, UVRAG				
Endosomal biogenesis	Sorting	ESCRT (ESCRT0, ESCRTI, ESCRTII and ESCRTIIII)				
	Early endosomes	Maker			Rab5a, b,c,d		
		Effector			APPL1/2, CORVET, EEA1, FHF complex, Huntingtin-HAP40, Mon1-Ccz1, PI-3-kinase, Rabankyrin, Rabaptin5, Rabenosyn		
		GTPase-activating protein			RabGAP-5		
	Late endosomes	Maker			Rab7		
		Effector			EPG5, FYCO, ORP1L, PLEKHM1, PI-3-kinase RILP, Rubicon, WDR91		
		GTPase-activating protein			TBC1D5, TBC1D15		

Currently, the majorly reported lysosomal transport pathways encompass autophagy, endocytosis, and phagocytosis. Lysosomes play a crucial role in autophagy. Autophagic transport pathways are cellular mechanisms that rely on lysosomal degradation to metabolize, clear, and recycle damaged proteins and organelles, thereby preserving cellular and metabolic homeostasis. These pathways encompass micro-autophagy, macro-autophagy, and chaperone-mediated autophagy.

Micro-autophagy: In micro-autophagy, cargo is directly sequestered onto lysosomes with the assistance of HSC70, ESCRT, and SNARE assembly. Following internalization via lysosomal membrane protrusions, invagination, or endosome invagination, the cargo undergoes degradation facilitated by lysosomal hydrolases (7).

Macro-autophagy: Macro-autophagy is responsible for degrading various subcellular materials and ubiquitin-labeled targets. During cellular stress, mTORC1 is inhibited, promoting the phosphorylation of the ULK1-FIP200-ATG13 initiation complex. This subsequently activates the Beclin-1-PI3KC3-VPS15/p150 complex, leading to phagophore elongation, which subsequently closes to form mature double-membrane-bound autophagosomes (12, 47–49). Autophagosomes are transported closer to lysosomes through the STX17-SNAP29-VAMP8 complex and recruit HOPS for membrane fusion with lysosomes, forming autolysosomes (50, 51). Within the autophagic flux process, cargo undergoes degradation and recycling.

Chaperone-mediated autophagy (CMA): Certain selected proteins containing KFERQ-like amino acid sequences or modified structures can also be targeted for degradation via CMA (52). KFERQ-like sequences facilitate protein binding to Hsc70, forming a complex with other co-chaperones such as HSP40, HIP, HOP, and HSP90. This complex then binds to lysosome-associated membrane protein 2A (LAMP2A) on lysosomal membranes (53). When the GXXG motif in the structural membrane remains intact, LAMP2A forms multimers, resulting in the formation of a translocation site that provides a transmembrane channel for the attached protein (52, 54).

The internalization of extracellular fluids or particles into cells via the formation of small or large vesicles derived from the plasma membrane is known as endocytosis and phagocytosis, respectively. These processes represent unique ways in which lysosomes contribute to lysosomal biogenesis (8, 55). Proteins originating from the plasma membrane or the trans-golgi network (TGN), such as M6PRs carrying lysosomal hydrolases, form early endosomes through the constitutive secretory pathway and are marked by Rab5 and EEA1 (56). During this process, lysosome-associated proteins from the endoplasmic reticulum (ER) can directly or indirectly join in (57). Early endosomes contain structures known as tubular sorting endosomes (TSE) that transport recycling proteins back to the plasma membrane or TGN (58). Meanwhile, ESCRT sorting mechanisms primarily guide the degradation of ubiquitin-tagged proteins in endosomes and regulate the formation of early endosomes, as exemplified by the EGFR (6, 59). Additionally, there exist ESCRT-independent sorting mechanisms that enrich proteins like CD63 and MHC-II in exosomes secreted into the extracellular space (60, 61). Mon1-Ccz1 and Rab11 mediate fusion and fission events, giving rise to late endosomes. Upon activation of the Rab7 GTPase enzyme, late endosome positioning is regulated through microtubule-dependent motor proteins RILP and FYCO1, and membrane fusion occurs through SNARE complexes, leading to heterotypic or homotypic fusion and the formation of lysosome-related organelles (57). Ultimately, this process results in the degradation of cargo within lysosomes. The relevant summary is shown in **Table 1**.

## 3 Lysosome-associated autophagy and tumor immunity

Autophagy is a process in which cellular contents are encapsulated in double-membrane vesicles and fused with lysosomes for degradation, which removes damaged or redundant organelles, proteins, and other materials from cells (62). The process of autophagy includes the formation of autophagosomes, the fusion of autophagosomes with lysosomes, and the degradation of autophagic substances in lysosomes. Lysosomes are the end point of autophagy and where autophagic substances are recycled or excreted from the cell (63). Autophagy and lysosomes have important effects on cellular metabolism, signal transduction, immune response, and death. The balance between autophagy and lysosomes is important for maintaining cellular homeostasis and coping with various stresses. Imbalance between autophagy and lysosomes may lead to various diseases such as neurodegenerative diseases, infectious diseases and tumors (64). Autophagy has a double-edged role in tumorigenesis and progression, inhibiting tumor formation as well as promoting tumor survival and drug resistance. Autophagy also affects tumor immunity in a variety of ways, including regulating tumor cell immunogenicity, antigen presentation, cytokine secretion, and immune checkpoint protein expression may be positive or negative, depending on the different cell types, tumor types, and treatment conditions (65).

Among the negative effects of autophagy on tumor immunity, on the one hand, autophagy reduces the expression of MHC-I molecules on the surface of tumor cells by targeting them to the lysosome for degradation or by blocking their assembly in the endoplasmic reticulum, thus reducing the visibility and sensitivity of tumor cells to CD8^+^ T cells (66). Alec C. Kimmelman et al. found that in pancreatic ductal adenocarcinoma (PDAC) cells, MHC-I molecules are selectively targeted to lysosomes for degradation rather than presenting antigen at the cell surface. This process is dependent on autophagy, specifically the autophagy cargo receptor NBR1, which binds to MHC-I and delivers it to autophagosomes and lysosomes. Knockdown or pharmacological inhibition of autophagy-related genes or proteins reduces the degradation of MHC-I in lysosomes and restores the level of MHC-I on the surface of PDAC cells. In an mouse model *in vivo*, autophagy inhibition reduces PDAC cell degradation in lysosomes and enhances PDAC cell killing by CD8^+^ T cells (16). On the other hand, autophagy can regulate the function of immune cells by affecting their differentiation, polarization, activation, migration, and other processes, thereby attenuating their killing effect on tumor cells or enhancing their promoting effect on tumor cells. Panayotis Verginis et al. found that lysosomes function in myeloid-derived suppressor cells (MDSCs) not only in degradation and recycling, but also in the regulation of signal transduction and gene expression (67). The mTORC1 is an important regulator of cell growth and metabolism. When the amino acid level in lysosomes is high, the receptor on the surface of lysosomes activates mTORC1 and promotes the proliferation and maturation of MDSCs. When autophagy is enhanced, the amino acid level in lysosomes decreases, leading to inhibition of mTORC1, which affects the function of MDSCs (67). Xu et al. found that in renal cell carcinoma (RCC) patients, high expression of LAPTM4B was associated with a significant increase in M2-type macrophages in the TME, which is thought to be associated with tumor metastasis. LAPTM4B is a lysosome-associated gene that regulates the number and function of lysosomes and affects the level and efficiency of autophagy, and it was found that LAPTM4B was expressed at different levels in different types of renal cell carcinoma cells and was associated with lysosomal and autophagic pathway activity. The positive effect of autophagy on tumor immunity can also be attributed to the modulation of immune cell function, which can weaken the killing effect of immune cells on tumor cells or enhance the promotion effect of immune cells on tumor cells (68). The role of autophagy in nuclear factor-kappa B (NF-κB) regulation was explored in a study by Lei et al. by using a macrophage differentiation model. The authors found that lysosome-dependent pathways lead to the translocation of NF-κB p65 from the nucleus to aggregates (ALS) and degradation through selective autophagy. The lysosomal inhibitor bafilomycin A1 prevented the degradation of NF-κB p65, allowing M2 macrophages to produce high levels of proinflammatory factors. In addition lysosomal dysfunction leads to accumulation of NF-κB p65 in ALS, increasing immunosuppression in M2 macrophages (69).

## 4 Lysosome-related metabolism and tumor immunity

### 4.1 Lysosomal lipid peroxidation

Lipid peroxidation is a biochemical reaction that refers to the formation of peroxides when unsaturated fatty acids or their derivatives lose hydrogen atoms in response to oxidative stress. Lipid peroxides are highly reactive and toxic and can damage cell membranes, proteins, DNA and other biomolecules (70). Lysosomes are intracellular digestive organs that degrade various biomolecules, including lipids. lysosomal lipid peroxidation (LLP) and cell death induced by the lysosomal inhibitor DC661 was found to be independent of known programmed cell death pathways (autophagy, apoptosis, necroptosis, iron-death, and inflammatory death) as well as iron, calcium, or proteolytic enzymes in lysosomes in a study by Ravi K. Amaravadi et al. LLP is a mechanism that drives lysosomal cell death and a unique form of immunogenic cell death (ICD). ICD refers to a mode of cell death that activates an anti-tumor immune response and is characterized by the surface or release of a number of immune-stimulating molecules, such as calreticulin (CRT), HMGB1, and ATP, from tumor cells. LLP-induced ICD enhances the immunogenicity of tumor cells, allowing them to be recognized and cleared by the immune system. LLP also activates terminally differentiated T cells and enhances T cell-mediated killing. Tumor cells treated with the LLP inducer DC661 can serve as an effective tumor vaccine, inducing an adaptive immune response and achieving tumor rejection in “immunologically hot “ tumors (71). Hence lysosomal inhibitors can be used as a novel ICD inducer that works synergistically with other immunotherapeutic tools, such as ICIs and CAR-T cells, to improve the efficacy and breadth of tumor immunotherapy.

### 4.2 Hypoxia and lysosomes

Hypoxia or inadequate tissue oxygenation is a critical factor in the difficulty of eradicating most cancers (72). In hypoxic conditions, tumor cells activate the hypoxia-inducible factor (HIF)-1α, which induces many downstream target genes, including vascular endothelial growth factor (VEGF), and promotes tumor angiogenesis, gene expression, exosome secretion, etc., resulting in stronger proliferation, invasion, and metastasis of tumor cells (73). The hypoxic microenvironment also affects the level of lysosomal function and thus tumor immunity. The research by Manran Liu et al. suggests that under hypoxic conditions in breast cancer’s Cancer-Associated Fibroblasts (CAFs), lysosomal dysfunction occurs where autophagosomes fail to fuse with lysosomes but instead merge with multivesicular bodies (MVBs), promoting exosome release and subsequently enhancing cancer cell invasion and metastasis (74). Specifically, the dysregulated ATM is oxidatively activated in CAFs and phosphorylates a key lysosomal proton pump, ATP6V1G1, leading to impaired lysosomal acidification. Concurrently, the oxidized ATM phosphorylates BNIP3, inducing autophagosome accumulation and exosome release. Additionally, Zhang et al. found that hypoxia significantly increases the number of extracellular vesicles (EVs) secreted by HNSCC cells (75). In a hypoxic environment, HIF1a negatively regulates the expression of a V-ATPase member, ATP6V1A, disrupting the acidic environment within lysosomal lumen and lysosomal integrity, thus reducing MVB and lysosome fusion and promoting the release of intraluminal vesicles as EVs (76). Tumor EVs can interact with immune cells, affecting immune responses. Tumor EVs surface PD-L1 can bind to CD8^+^ T cell surface programmed cell death protein 1 (PD-1), inhibiting CD8^+^ T cell activation and proliferation; surface ICAM-1 can interact with T cell surface LFA-1, promoting EVs PD-L1 mediated immune suppression; tumor EVs can also induce immune cell apoptosis or differentiation into immunosuppressive cells (77, 78). These studies indicate that hypoxia in tumor cells can affect lysosomal function and regulate EVs secretion, making lysosome-EVs a novel target for tumor immune therapy.

Additionally, HIF1a is a critical transcriptional regulator that enables metabolic adaptation in cells under low oxygen levels (79). The stability and activity levels of HIF1a are controlled by various mechanisms. Previous studies have discovered that HIF1a can bind to HSC70 and LAMP2A for transport and become destabilized in lysosomes via the CMA pathway (2). Using lysosomal inhibitors can rescue lysosomal expression of HIF1a in epithelial tumor cells. However, recent research indicates that in melanocytes, the degradation of HIF1a protein is not sensitive to chloroquine but instead is associated with the Golgi apparatus. Authors using Brefeldin A to disrupt endoplasmic reticulum-Golgi transport found that HIF1a is localized to the nucleus and its transcriptional activity is decreased. These results suggest that in cells undergoing different degradation pathways, there may exist several distinct pools of HIF1a (80). In tumor cells, HIF1a might act not only as a molecular sensor for oxygen levels but also to monitor protein-folding capacities, ensuring cancer cells adapt to hypoxic conditions and avoid the detrimental effects of low oxygen. Therefore, the relationship between HIF1a and various organelles remains complex and necessitates further, more definitive research.

### 4.3 Glucose metabolism and lysosomes in tumor immune cells

The relationship between lysosomes and glucose metabolism is a newly discovered area of research with important physiological and pathological implications. Lysosomes are not only able to utilize glucose as an energy source, but also sense glucose availability and regulate cell metabolism, growth and survival through signaling pathways (81). Cao et al.’s study found that M2-type TAMs had the highest individual uptake of intra-tumor glucose, and its increased glucose uptake promoted hexosamine biosynthesis pathway (HBP)-dependent O-GlcNAcylation modification, which enhanced the pro-cancer function of TAMs. Glucose metabolism mediates O-GlcNAcylation modification of Cathepsin B at the Ser210 site via lysosomal OGT and increases the level of mature Cathepsin B in macrophages and its secretion in the TME (82). Cathepsin B is a lysosomal protease that degrades the extracellular matrix and promotes tumor cell invasion and migration (83). This suggests that lysosomal OGT, a key molecule of glucose metabolism in regulating the pro-cancer function of TAM, affects lysosomal function and tumor biological behavior by modifying Cathepsin B via O-GlcNAcylation. This finding provides new insights and ideas for tumor immunotherapy.

## 5 Important influence of lysosomes on antigen presentation process

Antigen presentation refers to a process in the immune system that involves a number of APCs capturing, processing, and presenting antigenic fragments for recognition by T cells (84). Antigen presentation is categorized into two types, which are MHC-I and MHC-II (85). MHC is a polymorphic protein that binds antigenic fragments and transports them to the cell surface (86). MHC-I antigen presentation refers to the presentation of endogenous antigens, i.e., antigens originating from within the cell, such as viral or tumor proteins, by all nucleated cells (as well as platelets) via MHC-I molecules. These antigens are degraded by the proteasome into small peptide segments, which are then transported through the transporter protein associated with antigen presentation (TAP) to the ER, where they bind to MHC-I molecules and are transported to the cell surface. This approach allows CD8^+^ T cells to recognize and kill infected or mutated cells (85, 86). MHC-II antigen presentation refers to the presentation of exogenous antigens, i.e., antigens originating from outside the cell, such as bacteria or parasites, by some specialized APCs via MHC-II (87). These antigens, after being phagocytosed or endocytosed by APCs, are hydrolyzed into small peptide fragments in endosomes or lysosomes, which then bind to MHC-II molecules and are transported to the cell surface. This approach allows CD4^+^ T cells to recognize and provide assistance signals to other immune cells (88, 89).

Lysosomes provide raw material for antigen presentation by breaking down phagocytosed or endocytosed antigens into small peptide fragments via hydrolytic enzymes. Lysosomes play an important role in tumor immunity, on the one hand, they can participate in the MHC-I inhibitory axis of tumor cells, thus reducing the expression of MHC-I on the cell surface and allowing tumor cells to evade recognition and clearance by T cells. Jun Wang et al. identified a pan-tumor cell surface MHC-I inhibitory axis (STW axis) dominated by the membrane proteins SUSD6 and TMEM127. The two membrane proteins, SUSD6 and TMEM127, can bind to MHC-I and recruit the E3 ubiquitin ligase WWP2 to ubiquitinate MHC-I, which in turn promotes the endocytosis of MHC-I from the cell surface. After endocytosis, MHC-I is transported to early endosomes and then degraded in lysosomes by fusion with lysosomes. And the lysosomal inhibitor chloroquine inhibits SUSD6 and TMEM127-mediated MHC-I degradation, thereby increasing MHC-I expression on the cell surface (87). Li et al. also found that PCSK9 physically binds to MHC I and promotes its degradation in lysosomes, thereby reducing its recycling and reutilization on the cell surface, and confirmed that the degradation of MHC-I by PCSK9 occurs via the lysosomal pathway using drug intervention experiments (90). Keisuke Yamamoto et al. found that MHC-I in PDAC cells is mainly located in autophagosomes and lysosomes rather than on the cell surface (16). MHC-I is recognized by the autophagy receptor NBR1 and selectively transported to lysosomes for degradation via the autophagy pathway. Inhibition of autophagy or lysosomes restores MHC-I expression on the surface of PDAC cells, which enhances antigen presentation, activates CD8^+^ T cells and DCs, and inhibits tumor growth (16).Zou et al. found that optineurin deficiency impaired the integrity of the IFN-γ and MHC-I signaling pathways through palmitoylation-dependent IFNGR1 lysosomal transport and degradation, which drove immune escape and endogenous immunotherapy resistance in rectal cancer (91). On the other hand, lysosomes can promote tumor cell MHC-II antigen presentation, thereby enhancing the effects of immune surveillance and immune checkpoint blockade therapy. Merkel cell carcinoma (MCC) is a rare and highly aggressive neuroendocrine skin cancer, with most cases associated with Merkel cell polyomavirus (MCPyV) infection (92). Teri Heiland et al. showed that a cancer vaccine was designed to promote a potent, antigen-specific CD4^+^ T-cell response to MCPyV-LT. The authors utilized their nucleic acid platform UNITE™ (UNiversal Intracellular Targeted Expression), which fuses tumor-associated antigens to LAMP1 (93). LAMP1 is a glycoprotein localized on lysosomal and endosomal membranes that transports antigen from the cytoplasm to the lysosome, thereby enhancing antigen presentation on MHC-II (94). This lysosomal targeting technology enhances antigen presentation and balanced T-cell responses. LAMP1-MCPyV-LT induced stronger CD4^+^ T cell responses and higher levels of anti-MCPyV-LT antibodies in mice compared to MCPyV-LT without LAMP1 fusion (93). Yifan Ma et al. designed a self-adjuvanted nanovaccine (SANV) in a study that constructed an individualized tumor immunotherapy strategy using a pH-sensitive galactosylated dextran-retinoid (GDR) nanogel as a carrier and the patient’s own tumor cell lysates as antigens. The nanogel is pH-sensitive and capable of releasing antigens and retinoids in acidic lysosomes, the latter of which activates the retinoic acid receptor (RAR) signaling pathway and promotes DCs maturation. In addition, it induces lysosomal cleavage, leading to intracellular reactive oxygen species (ROS) production, which enhances proteasome activity and MHC-I molecule-mediated antigen cross-presentation, activates the CTL response, and inhibits the differentiation and function of Tregs and TAMs (95).

Furthermore, lysosomes regulate the efficiency and quality of antigen presentation, by controlling the extent and timing of antigen degradation, as well as influencing the stability and expression levels of MHC molecules. A study conducted by Sanjay Garg et al. designed a novel microencapsulated cancer vaccine (egg white-loaded pH/redox dual-sensitive microencapsulated vaccine, OLM-D) that combines an antigen with a homemade amphiphilic poly(l-histidine)-poly(ethylene glycol) (PLH-PEG) via cleavable covalent binding. Lysosomal escape is the process by which endocytosed material is released from the lysosome into the cytoplasm, which enhances antigen processing and presentation within the cell. OLM-D rapidly releases egg white under acidic or reducing conditions, leading to lysosomal rupture and ROS production, which triggers lysosomal escape. OLM-D enhances antigen processing and presentation by DCs through lysosomal escape, thereby activating the immune response of T cells (96).

## 6 The crucial impact of lysosomes on immune checkpoint molecules

Immune checkpoints are certain molecules that can regulate the activation and function of immune cells, maintaining immune homeostasis and self-tolerance (97). Several immune checkpoint molecules, such as PD-L1, can be present on the cell surface or intracellularly and inhibit T cell responses by binding to receptors. There are interactions and regulation between immune checkpoint molecules and lysosomes (98). On one hand, lysosomes can regulate their expression levels and functions by degrading immune checkpoint molecules. On the other hand, immune checkpoint molecules can also influence the formation and function of lysosomes. There exists a dynamic balance and mutual regulation between immune checkpoint molecules and lysosomes, which is significant for the proper functioning of the immune system and cancer immunotherapy (99, 100).

### 6.1 PD-1 and PD-L1

PD-L1 is an immune checkpoint molecule that can bind to the PD-1 receptor, inhibiting the activation and proliferation of T cells, thereby allowing tumor cells to evade immune surveillance (101). Antibodies targeting PD-L1 can disrupt the interaction between PD-L1 and PD-1, activating T cells to exert cytotoxic effects on tumors. This is an effective cancer immunotherapy approach. However, PD-L1 is not only present on the cell surface but also within cells, with its expression levels dynamically regulated through dynamic transport and degradation mechanisms (102). Therefore, merely blocking the surface molecules of PD-L1 with antibodies may not be sufficient to fully inhibit PD-L1’s function. PD-L1 is expressed on the cell membrane but can also be internalized and transported to lysosomes for degradation. The degradation process involves various molecular modifications, including glycosylation, phosphorylation, and palmitoylation (99, 102).

A study by Xu et al. discovered a novel mechanism regulating PD-L1 expression and function, namely, palmitoylation modification. Palmitoylation is a lipid modification, which refers to the covalent attachment of palmitic acid to cysteine residues on proteins via a thioester bond. This modification alters the protein’s hydrophobicity, stability, subcellular localization, and function, allowing the protein to anchor to the cell membrane (103). The authors demonstrated the presence of a palmitoylation site in the cytoplasmic domain of PD-L1, which can undergo palmitoylation modification catalyzed by the enzyme DHHC3. This modification inhibits the ubiquitination of PD-L1, thereby preventing its degradation in the lysosome, enhancing the stability and function of PD-L1. Inhibiting the palmitoylation of DHHC3 or PD-L1 through pharmacological or genetic methods can reduce the expression of PD-L1 in tumor cells, enhancing the cytotoxic effect of T cells against the tumor. The authors also designed a competitive inhibitor that can selectively target the palmitoylation site of PD-L1, thereby inhibiting the expression and function of PD-L1 and enhancing the immune response of T cells against tumors. Hence, lysosomes play a crucial role in regulating the expression and function of PD-L1 (102).

The glycosylation of PD-L1 on the cell membrane is a crucial regulatory factor for its stability and function, affecting the affinity and signal transduction between PD-L1 and PD-1 (104, 105). Zhou et al.’ research also investigated how glycosylation modifications of programmed cell death ligand 2 (PD-L2) promote immune evasion and resistance to anti-EGFR therapy in tumor cells (106). PD-L2 is an immune checkpoint molecule capable of binding to PD-1, and its expression on the surface of tumor cells can inhibit the activity and function of T cells (107, 108). The authors have discovered a novel enzyme, FUT8, which can perform N-glycosylation modifications on PD-L2 and enhance its binding to EGFR. Glycosylation-modified PD-L2 activates the EGFR/STAT3 signaling pathway while reducing the affinity of cetuximab for EGFR. Glycosylation modifications also stabilize the protein levels of PD-L2 by blocking the ubiquitin-dependent lysosomal degradation pathway, thereby enhancing the binding of PD-L2 to PD-1 and the immune evasion effect (106).

The phosphorylation of PD-L1 is a crucial step in its intracellular trafficking and degradation, involving various kinases and phosphatases (109). The known phosphorylation sites of PD-L1 currently include S195, S197, and Y248. The phosphorylation site primarily involved in the lysosomal degradation pathway is S197 (S195 phosphorylation site is associated with PD-L1 degradation pathway dependent on the endoplasmic reticulum, while Y248 is located in the extracellular region of PD-L1, primarily enhancing the affinity and signal transduction between PD-L1 and PD-1) (109). GSK3β is a multifunctional kinase that can participate in various signaling pathways and biological processes (110). The phosphorylation of PD-L1 by GSK3β or AMPK promotes its translocation from the cell membrane to lysosomes. Therefore, GSK3β or AMPK can reduce the stability and function of PD-L1 on the cell membrane in this manner, enhancing T cell-mediated cytotoxicity against tumor cells (111).

**Figure 1:**
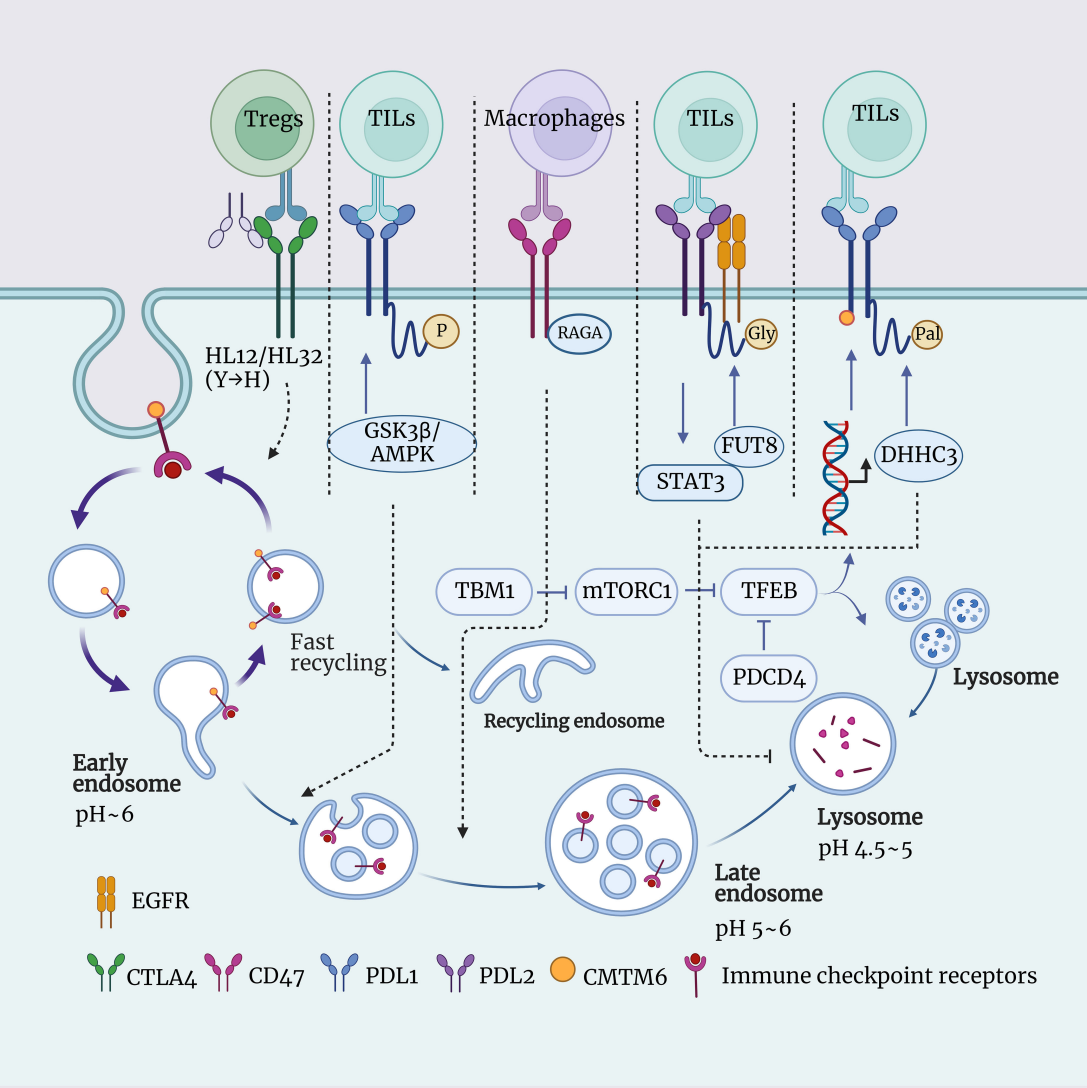
Schematic representation of the interaction between lysosomes and immune checkpoints. This figure illustrates the role of lysosomes in immune checkpoint signaling, including CTLA4, PDL1, PDL2, CD47, and more. Lysosomes are intracellular digestive organelles capable of degrading materials from phagocytosis or autophagy. Lysosomes also have the ability to modulate the expression and function of immune checkpoints, thus influencing the balance of immune responses. The aim is to highlight the significant role of lysosomes in immune checkpoint signal transduction and their potential as therapeutic targets.

Additionally, a study by Mark A. Dawson et al. also found the significant role of lysosomes in the degradation of PD-L1. They utilized CRISPR-Cas9 technology to perform a whole-genome knockout screen in the pancreatic cancer cell line BxPC-3 and discovered an unknown protein, CMTM6. CMTM6 is a recently discovered regulator of PD-L1 expression. It does not affect the maturation of PD-L1 but co-localizes with PD-L1 on the cell membrane and recycling endosomes, preventing PD-L1 from being transported to lysosomes for degradation, thus maintaining its stability on the cell surface (18, 112). Additionally, a study by Zhang et al. investigated how targeting the free fatty acid receptor 4 (GPR84) in MDSCs can overcome resistance to PD-1 immune therapy in esophageal cancer (113). These studies have all revealed the crucial role of the lysosomal degradation pathway of PD-L1 in regulating immune evasion by tumor cells, providing new targets and mechanisms for the development of novel immunotherapy strategies. On the other hand, immune checkpoint molecules can also influence the formation and function. A study by Deng et al. found that tubeimoside-1 (TBM-1) can significantly reduce the expression of PD-L1 on the surface of various cancer cells (113). TBM-1 can activate TFEB, a key regulator of lysosome biogenesis, by inhibiting the activation of mTORC1. TFEB can transcriptionally activate various lysosome-related genes, including LAMP1, LAMP2, CTSD, etc., thereby increasing the quantity and functionality of lysosomes (114). TFEB can also promote the lysosomal degradation of PD-L1 by binding to the promoter region of its encoding gene CD274, inhibiting its transcription. In this way, TFEB can reduce the surface levels of PD-L1 on cancer cells, enhancing the recognition and killing of cancer cells by immune cells. In addition, TBM-1 can also enhance the TME through TFEB, increasing the quantity and activity of TILs while reducing the number and functionality of immunosuppressive cells (MDSCs and Tregs). These effects are associated with the degradation of PD-L1 by TBM-1, as the impact of TBM-1 on the TME disappears in PD-L1 knockout cancer cells (113) (**Figure 1**).

### 6.2 Cytotoxic t lymphocyte-associated protein-4

CTLA-4 is another common immune checkpoint molecule that regulates the activity of T cells by binding to CD80/CD86, preventing the occurrence of autoimmune diseases (115). The expression of CTLA-4 on the surface of T cells is dynamically regulated. It can be internalized into intracellular vesicles through endocytosis, and then it can either return to the cell surface through recycling or be sent to lysosomes for degradation (116, 117). When CTLA-4 is transported to the lysosome, it is hydrolyzed by enzymes into smaller fragments, thereby reducing the quantity of CTLA-4 on the surface of T cells, which impacts the function of T cell (118). The anti-CTLA-4 monoclonal antibody (mAb) is a means of cancer immunotherapy that can enhance the cytotoxicity of T cells against tumor cells by blocking the interaction between CTLA-4 and CD80/CD86 (119). The anti-CTLA-4 mAb currently used in clinical practice, such as Ipilimumab and Tremelimumab, all lead to the degradation of CTLA-4 in lysosomes, thereby reducing the function of Tregs and increasing the risk of immune-related adverse events (irAEs). However, this also results in an improvement in cancer immunotherapy effectiveness (119, 120). Yang Liu et al.’ research has identified a novel anti-CTLA-4 mAb, HL12 or HL32. These antibodies can dissociate from CTLA-4 after endocytosis and, through an LRBA-dependent mechanism, facilitate the recycling of CTLA-4 to the cell surface. This, in turn, preserves the functionality of Treg cells, reducing the occurrence of irAEs, but simultaneously limiting the improvement in cancer immunotherapy effectiveness. Furthermore, to enhance the bioavailability of antibodies, the authors introduced a mutation from histidine (H) to tyrosine (Y). This mutation increased the pH sensitivity of the anti-CTLA-4 mAbs, allowing it to dissociate from CTLA-4 in acidic environments, thus avoiding lysosomal degradation. These pH-sensitive anti-CTLA-4 mAbs have been shown to more effectively deplete Tregs within tumors and eradicate established tumors (120). This is a new paradigm in cancer research, wherein altering the pH sensitivity and recyclability of anti-CTLA-4 mAbs through lysosomal characteristics can simultaneously enhance its safety and efficacy (**Figure 1**).

### 6.3 Others

Programmed cell death factor 4 (PDCD4) is a tumor suppressor associated with cell cycle and apoptosis, capable of modulating various cellular processes such as autophagy, inflammation, transformation, and invasion by inhibiting the function of eukaryotic initiation factor 4A (eIF4A), thereby influencing protein translation (121). PDCD4 is downregulated or lost in various tumors and is associated with the occurrence, progression, and prognosis of cancer (122). The research by Zhang et al. found that PDCD4 can reduce the overall levels of TFEB, thereby inhibiting its accumulation and transcriptional activity within the cell nucleus, ultimately leading to a decrease in the number and function of lysosomes (123). TFEB is a transcription factor that regulates various cellular processes, including lysosome biogenesis, autophagy, lysosomal exocytosis, lipid metabolism, and energy metabolism. Its activity is tightly controlled by multiple post-translational modifications, protein interactions, and spatial distribution (124). And it has been demonstrated that the inhibition of lysosome function by PDCD4 depends on TFEB, and in the TME, PDCD4 deficiency can promote the anti-tumor effect of macrophages by enhancing TFEB expression (123) (**Figure 1**).

CD47 is an immune checkpoint, which is a molecule capable of regulating immune responses (125). CD47 primarily inhibits the phagocytosis of tumor cells by macrophages by binding to the surface receptor SIRPα and sending a “don’t eat me” signal (125). In this way, tumor cells can evade the clearance of the immune system, leading to immune escape. Therefore, drugs targeting CD47 or SIRPα can block this signal, restoring the phagocytic function of macrophages, thereby achieving anti-tumor effects (19). Currently, there are various forms of drugs under development, including CD47 antibodies, SIRPα fusion proteins, SIRPα antibodies, and CD47 bispecific antibodies, among others. These drugs can not only enhance the phagocytosis of tumor cells by macrophages but also activate other immune cells such as T cells and NK cells, inducing apoptosis in tumor cells, and more (19, 126). The research by Jin et al. found that RAGA can bind to CD47 and promote the transport of CD47 from late endosomes to lysosomes for degradation (20). The downregulation of RAGA leads to the accumulation of CD47 on the surface of LUAD cells, enhancing the binding of CD47 to SIRPα and inhibiting macrophage phagocytosis of LUAD cells (20). The current relationship between CD47 and lysosomes has not been fully elucidated. However, in the future, by exploring the impact of CD47 degradation in lysosomes on other signaling pathways and biological functions, the development of interventions targeting the CD47 lysosomal degradation process holds significant promise for enhancing the efficacy of cancer immunotherapy (**Figure 1**).

## 7 Lysosomal effects on different immune cells in the TME

### 7.1 TAMs

TAMs are the most abundant immune cells in the TME and can be classified into M1 and M2 types based on their function and phenotype (127). M1 macrophages exhibit anti-tumor activity, secreting pro-inflammatory cytokines and cytotoxic molecules, activating T cells and natural killer cells. M2 macrophages, on the other hand, promote tumor growth, secretion of anti-inflammatory cytokines and growth factors, facilitating tumor growth, metastasis, angiogenesis, and immune evasion (128).

Lysosomes play a crucial role in regulating the polarization and function of TAMs. On one hand, lysosomes can influence the signaling pathways of TAMs, thereby affecting their functional outputs. Wei et al.’ research revealed that LAMP2a is upregulated in TAMs and plays a significant role in tumor progression (129). Inducing LAMP2a inactivation through shRNA or CRISPR/Cas9 can impede TAMs activation and tumor growth. LAMP2a degradation leads to the promotion of tumor-activating macrophages by degrading peroxiredoxin 1 (PRDX1) and CREB-regulated transcription coactivator 1 (CRTC1). In addition, extracellular vesicles released by tumor cells (T-MPs) can also influence macrophage polarization by affecting the characteristics of lysosomes (129). Lysosomes are vesicles containing acidic hydrolytic enzymes that can degrade engulfed substances. When T-MPs enter lysosomes, they disrupt lysosomal function, leading to an increase in lysosomal pH and the release of calcium. The molecular events enable T-MPs to induce the transformation of M2 macrophages into M1 type through the lysosome-dependent pathway. The DNA signals carried by T-MPs can activate the cGAS-STING-TBK1-STAT6 signaling pathway within macrophages, thereby inducing macrophages to express anti-inflammatory factors such as IL-10 and TGF-β, forming M2-type macrophages (129). However, when T-MPs are delivered into lysosomes, they release DNA fragments and bind with cGAS, thereby activating the STING-TBK1-IRF3 signaling pathway, inducing macrophages to express pro-inflammatory cytokines such as IFN-β and TNF-α, leading to the formation of M1-type macrophages. Furthermore, the long-chain non-coding RNA signals carried by T-MPs can also induce macrophages to release pro-inflammatory cytokines, such as IL-1β, leading to the formation of M1-type macrophages. This effect is mediated by the long-chain non-coding RNA within T-MPs binding to TLR7 or TLR8 and being recognized and activated within lysosomes (129).

On the other hand, lysosomes can influence the metabolic state of TAMs, thereby affecting their phenotypic transition. Fan et al.’ research found that TFEB upregulates the expression of cytokine signaling suppressor protein 3 (SOCS3) and peroxisome proliferator-activated receptor gamma (PPARγ), as well as autophagic/lysosomal activity. This inhibition suppresses the NOD-like receptor family pyrin domain-containing 3 (NLRP3) inflammasome and HIF-1α-mediated hypoxic responses, thereby inhibiting a range of effector molecules in TAMs, including arginase-1, interleukin IL-10, IL-1β, IL-6, and prostaglandin E2 (130). The research has identified that TFEB is the primary regulatory factor of TAMs in breast cancer. TFEB controls TAMs’ gene expression and functions through various autophagy/lysosome-dependent and non-dependent pathways (130).

Furthermore, TAM can activate CD8^+^ T cells by cross-presenting antigens, thereby effectively eliminating tumors (131, 132). However, the cross-presentation ability of TAM is inhibited by the overactive cysteine proteases within its lysosomes, resulting in the inability to activate CD8+ T cells (133, 134). A study by Lev Becker et al. developed a DNA nanodevice (E64-DNA) that can target the lysosomes of mouse TAMs, inhibiting cysteine proteases within them, thereby enhancing TAMs cross-presentation capabilities, boosting CD8^+^ T cell responses, and cytotoxicity. E64-DNA can also alter the phenotype and function of TAMs, shifting them from the tumor-promoting M2 type to the tumor-inhibiting M1 type (133).

Multiple Myeloma (MM) is a malignant hematological disease caused by the clonal proliferation of plasma cells. The occurrence and development of MM are closely associated with immune cells in the TME, among which TAMs are one of the main cell types (135). The NLRP3 inflammasome is a multiprotein complex composed of NLRP3, apoptosis-associated speck-like protein containing a CARD (ASC), and caspase-1. It can recognize various endogenous and exogenous danger signals, activate caspase-1, and promote the maturation and secretion of IL-1β and IL-18 (136, 137). Beta-2 microglobulin (β2m) is a small molecular protein that primarily exists as the light chain of MHC-I molecules on the surface of all nucleated cells (136). In MM, elevated β2m levels are associated with tumor burden, renal impairment, and poor prognosis (138, 139). In a study conducted by Heiko Bruns et al., it was found that β2m can be engulfed by TAMs, leading to the formation of β-amyloid-like fibers within lysosomes. These β-amyloid-like fibers can trigger lysosomal rupture, releasing NLRP3 and ASC from within the lysosome. After lysosome rupture, NLRP3 and ASC can bind to caspase-1 in the cytoplasm, forming the NLRP3 inflammasome. The NLRP3 inflammasome can activate caspase-1, promoting the maturation and secretion of IL-1β and IL-18 (136).

**Figure 2:**
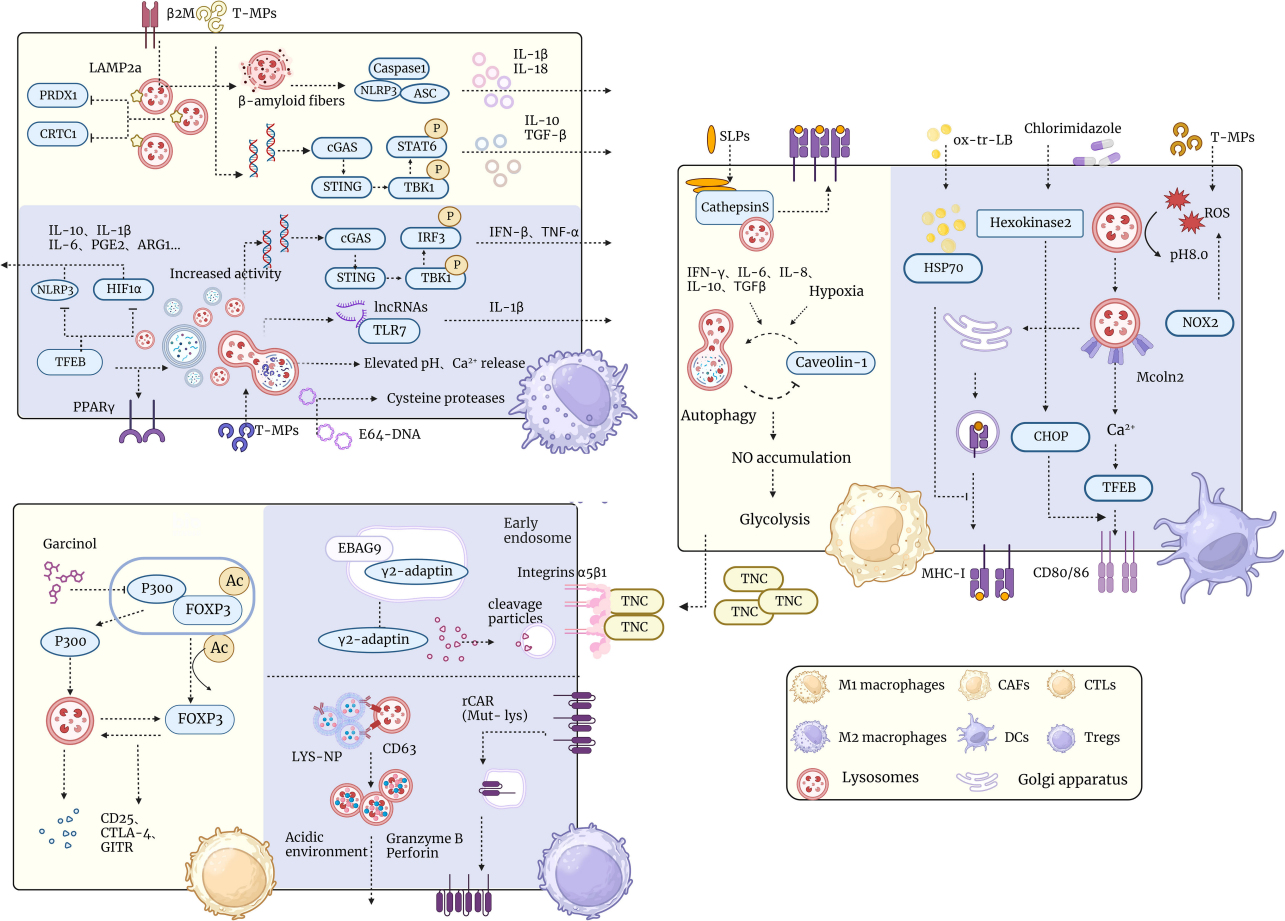
Schematic representation of lysosomal interactions with tumor microenvironmental immune cells. The diagram demonstrates the relationship of lysosomes in the tumor microenvironment with different types of immune cells such as TAM, T cells, CAF, DC cells, etc. Lysosomes have multiple functions in the tumor microenvironment, such as regulating immune cell polarization and activation, influencing tumor cell metabolism and proliferation, and participating in tumor-associated inflammation and immune escape. The diagram shows a number of lysosome-associated factors and pathways that can affect the functions and interactions of immune cells in the tumor microenvironment through different mechanisms, such as promoting or inhibiting immune responses and altering the phenotype and plasticity of tumor cells. This diagram aims to illustrate the important role of lysosomes in the tumor microenvironment and potential therapeutic targets.

The relationship between lysosomes and TAMs is complex and bidirectional, with different lysosomal functions potentially having different effects on TAMs. Modulating lysosomal function may be an effective strategy for regulating TAMs’ phenotype and function, thereby achieving tumor immunotherapy (**Figure 2**).

### 7.2 Relationship between lysosomes and T cell activation in the TME

T cells are crucial immune cells capable of recognizing and eliminating infected or mutated cells, including cancer cells (140). However, cancer cells can evade or inhibit T cell attack through a variety of mechanisms, resulting in a microenvironment conducive to tumor growth and metastasis (141). In the TME, T cells can be classified into different subpopulations based on their function, phenotype, and differentiation status, each having distinct roles and fates (142). In general, T cells in the TME can be divided into two major categories: tumor-specific T cells and non-tumor-specific T cells. Tumor-specific T cells refer to T cells capable of recognizing tumor antigens and initiating an immune response against them, including CD8^+^ cytotoxic T cells and CD4^+^ helper T cells (143). Non-tumor-specific T cells refer to T cells that cannot recognize tumor antigens or are unresponsive to them, including Tregs, double-negative (DN) T cells, gamma-delta (γδ) T cells (141, 144, 145). The non-tumor-specific T cells typically possess immunosuppressive or regulatory functions, which can inhibit the activity or proliferation of tumor-specific T cells.

The functionality of TILs may be influenced by their impact on the endolysosomal pathway’s biosynthesis and membrane fusion processes. The formation and function of lysosomes are closely related to endosomes. Endosomes are vesicular structures involved in the process of endocytosis. They can envelop extracellular substances or liquids into cells. Subsequently, they fuse with lysosomes to form endolysosomes, where hydrolytic degradation takes place (146). The research by Armin Rehm et al. discovered that Estrogen receptor-binding fragment-associated gene 9 (EBAG9) is an intracellular membrane protein that can bind with γ2-adaptin to form an intracellular membrane complex (147). The binding of EBAG9 to γ2-adaptin inhibits the recognition and binding of γ2-adaptin to the progranulin precursor protein, thereby reducing the transport efficiency of the progranulin precursor protein. In the absence of EBAG9 in Cytotoxic T Lymphocytes (CTLs), the lytic granules become smaller, possibly due to the interaction between EBAG9 and γ2-adaptin, which also affects the membrane fusion process of lysosomes, thereby regulating the adaptive immune response function mediated by CTLs (147).

Tregs are a type of lymphocyte that can suppress immune responses and play a crucial role in maintaining immune tolerance and preventing autoimmune diseases (148, 149). However, Treg cells can also suppress anti-tumor immunity, thereby promoting tumor growth and metastasis (150). Hence, it is an important scientific question how to effectively regulate the activity of Treg, both to treat autoimmune diseases and to enhance anti-tumor immunity. Forkhead box p3 (FOXP3) is a transcription factor that regulates Treg development and function (151). Zhang et al. found that the natural p300 inhibitor Garcinol dissociates p300 from the FOXP3 complex and undergoes lysosome-dependent degradation (152). Interaction between p300 and FOXP3 promotes their lysosomal transport and degradation. When p300 is inhibited by Garcinol, it dissociates from the FOXP3 complex and is delivered to the lysosome for hydrolysis. This leads to a decrease in the level of FOXP3 acetylation, which allows FOXP3 to be degraded by the lysosome as well. This process could not be rescued by the proteasome inhibitor MG132, suggesting that the lysosome is the major pathway for p300 and FOXP3 degradation. Garcinol is able to affect the function of Treg cells by inhibiting p300 and decreasing the level of FOXP3 acetylation. Garcinol is able to reduce the inhibitory effect of Tregs on effector T cells (Teff) by decreasing the expression of some key molecules by Treg cells, such as CD25, CTLA-4, and glucocorticoid-induced TNF receptor (GITR). In addition, Garcinol was able to increase the expression of some pro-inflammatory factors such as IL-17 and IFN-γ by Tregs, thus enhancing the killing effect of Tregs on tumor cells (152). Accordingly, it is possible that targeting lysosomes could limit Treg function and enhance the efficacy of tumor-targeted therapies.

### 7.3 DCs

DCs are specialized antigen-presenting cells capable of presenting foreign or self-antigens to T cells to initiate and regulate the immune response (153). There is a close relationship between DC cells and lysosomes. above all, lysosomes are important sites for antigen processing and presentation by DC cells. DC cells transport antigen to the lysosome by phagocytosis, autophagy, or cross-presentation, where hydrolysis, modification, and loading of the antigen onto MHC molecules occurs to form MHC-antigen peptide complexes, which are then transported to the cell surface via vesicles and presented to T cells (154). Huang et al. found that T-MP contain tumor antigenic profiles and innate signals, and that after endocytosis of T-MP by DC, T-MP is transported to the lysosome, where RO production catalyzed by NADPH oxidase 2 (NOX2) peaks the lysosomal pH from 5.0 to 8.5. This increase in pH, coupled with T-MP-driven lysosomal migration to the center, promotes the formation of MHC I-tumor antigen peptide complexes (155). Peter Cresswell et al. found that TNF-α, CpG, and LPS-induced cross-presentation of mature DCs was significantly reduced, whereas CD40L-induced cross-presentation of mature DCs was maintained relatively (156). This difference could not be explained by a decrease in antigen uptake or translocation to the cytoplasm, but was associated with an increase in endosomal/lysosomal acidification. In addition, the authors found that inhibition of endosomal/lysosomal acidification restored or enhanced cross-presentation of mature DCs (157). Thereby confirming that endosomal/lysosomal acidification is an important factor in regulating the cross-presentation capacity of mature DCs.

Additionally, lysosomes are critical regulators of DCs maturation and activation. DC cells have efficient antigen uptake but inefficient antigen presentation in the immature state DC cells have efficient antigen uptake but inefficient antigen presentation in the immature state. When DC cells encounter hazardous signals, maturation and activation occur, expressing high levels of MHC molecules and co-stimulatory molecules that enhance antigen presentation. Lysosomes play an important role in this process, and Bo Huang et al. found that T-MP activates Mcoln2 channels in lysosomes, releasing calcium ions, which in turn activates TFEB transcription factors, promoting the expression of CD80 and CD86, and enhancing DCs maturation and activation (158). Xia et al. found that clomidazole, an antifungal drug, promotes DC-mediated antigen presentation and enhances T-cell responses. Chlormidazole acts on hexokinase 2, regulates lactate metabolites, and enhances lysosomal pathway and Chop expression in DCs, thereby inducing DCs maturation and T cell activation (159).

Lysosomes are potential targets for immune tolerance and immunoresistance in DC cells. DC cells are immune-tolerant or immune-resistant in certain situations, such as TMEs, autoimmune diseases, etc. losing the ability to effectively activate T cells (160). DCs associated with tumors have been reported to be defective in ability to cross-present antigens (161). This is associated with abnormalities in lysosomal function. Dmitry I. Gabrilovich et al. found a high accumulation of liposomes (LBs) containing oxidatively truncated (ox-tr) lipids in tumor-associated DCs, but not in normal DCs. These ox-tr-LB are mainly derived from exosomes released by tumor cells after apoptosis. The ox-tr lipid in ox-tr-LB was able to bind to HSP70 via covalent bonding, thus blocking the interaction of HSP70 with the peptide-MHC I complex (pMHC). This leads to accumulation of pMHC in late endosomes/lysosomes rather than translocation to the cell surface. Consequently, tumor-associated DCs do not efficiently present antigen to CD8+ T cells, which reduces the intensity and quality of T cell responses (161). Maria G. Masucci et al. used a candidate specific antibody (idiotype) vaccine, IGKV3-20, as a model in their study, fusing it to the glycine-alanine repeat sequence (GAr) of the Epstein-Barr virus nuclear antigen (EBNA)-1 to inhibit its degradation in the proteasome and target it to lysosomal for processing. The fusion-type IGKV3-20 was more stable in mDCs and localized more to the lysosome than wild-type IGKV3-20. Transduction of fusion-type IGKV3-20 by mDCs efficiently induces CD4^+^ and CD8^+^ CTL responses, and these CTLs are able to kill autologous mother cells expressing IGKV3-20 or pulsed IGKV3-20-synthesized peptides or HLA-matched IGKV3-20-positive tumor cell lines (162). Hence our reasoning that fusion of a candidate specific antibody vaccine with GAr could broaden the immune response by facilitating the presentation of antigenic epitopes that require lysosome-dependent processing steps (**Figure 2**).

### 7.4 CAFs

CAFs are a distinct type of stromal cell found within the TME, acting as a supportive scaffold for the extracellular matrix of TME cells (163). In addition, CAFs secrete cytokines (such as IL-6, IL-8, IL-10, and IFN-γ), growth factors (like basic fibroblast growth factor (FGFβ) and TGFβ2), chemokines, and extracellular matrix proteins [such as tenascin-c (TNC)], which regulate angiogenesis, lymph-angiogenesis, and immune responses (163). Cytokines including IFN-γ, IL-6, IL-8, and IL-10, along with TGF-β, can induce autophagy in CAFs (164) Under chronic hypoxia, CAFs utilize autophagy to degrade caveolin-1. The reduced expression of caveolin-1 positively feeds back to drive autophagy upregulation, leading to NO accumulation, causing CAFs to adopt a glycolytic phenotype, producing a large amount of lactate, dynamically co-evolving with cancer cells, and becoming metabolically coupled (2, 165). ECM proteins from CAFs, notably TNC, interact with α5β1 integrins on the surface of T cells, inhibiting the reorganization of the actin cytoskeleton necessary for T cell activation, thereby preventing T cell proliferation and activation, and ultimately overcoming immune surveillance (166). TNC is degraded via the Skp2-p62 dependent autophagy-lysosomal system. In TNBC cells with impaired autophagy, accumulated TNC can lead to resistance against T cell-mediated immune attacks. Utilizing anti-TNC antibodies makes cells more sensitive to T cell-mediated tumor killing and enhances the anti-tumor efficacy of PD-1 blockade in autophagy-deficient TNBC tumors (32). Additionally, CAFs can suppress tumor-specific T cell functionality by expressing various immune-suppressive molecules (such as PD-L1, IDO, and TGF-β) or through the cross-presentation of exogenous antigens (163, 167). In research by Els ME Verdegaal and others, human colorectal cancer (CRC)-derived CAFs were used to investigate their capacity for cross-presenting novel antigen-derived synthetic long peptides (SLPs), i.e., tumor-derived mutated peptides, and the impact of this on tumor-specific T cell functionality (168). Human CRC-derived CAFs possess a greater capability to cross-present novel antigen-derived SLPs compared to normal colonic fibroblasts. The presentation of antigens by fibroblasts involves the lysosomal protease Cathepsin S. Lysosomes contain various hydrolases, among which Cathepsin S is a cysteine protease capable of cleaving proteins to generate peptides suitable for binding to HLA-I molecules. Cathepsin S is highly expressed in CAFs within human CRC tissues, enabling them to effectively cross-present novel antigen-derived SLPs, thereby inducing the activation of tumor-specific T cells (169).

## 8 The utilization of the acidic properties of lysosomes for the action of artificially synthesized drugs

We know that the regulation of TME is considered a method to enhance the effectiveness of cancer treatment. During the process of tumor drug therapy, the main obstacles leading to low drug delivery efficiency are the premature leakage of drugs and low cellular uptake efficiency. Therefore, based on the low pH characteristics of lysosomes, some novel nano/micromaterials have been designed to target lysosomes to enhance immunotherapy.

Wang et al. used lipid-coated calcium phosphate (LCP) nanoparticles (NPs) to co-deliver TRP2 mRNA encoding cancer antigen and PD-L1 siRNA to dendritic cells in lymph nodes. After internalization by dendritic cells, the dissolution of the NP core in the acidic compartment of lysosomes results in high osmotic pressure, thereby disrupting endosomal membranes to achieve effective endosomal escape and nucleic acid release. This promotes antigen presentation by dendritic cells and induces robust antigen-specific activation and proliferation of CD4^+^ and CD8^+^ T cells (13). VEGF and placental growth factor (PLGF) are two important pro-angiogenic factors that are overexpressed in breast cancer cells and M2-type TAMs. They promote tumor growth, metastasis, and immune suppression either synergistically or independently (170–172). Yin et al. developed a dual pH-sensitive multifunctional NPs for simultaneous delivery of VEGF siRNA and PLGF siRNA to M2-TAMs and breast cancer cells, achieving gene silencing of VEGF and PLGF. These NPs are composed of polyethylene glycol (PEG) and mannose dual-modified trimethyl chitosan (PEG-MT) and citraconic anhydride-grafted poly (PC). They possess characteristics such as prolonged circulation time in the bloodstream, enhanced accumulation in tumor tissues, active and passive targeting of M2-TAMs and breast cancer cells, endosomal/lysosomal escape, and intracellular siRNA release. These NPs exhibit a significant charge conversion under pH 5.0 and 6.5 conditions, promoting endocytosis and endosomal/lysosomal escape. This process is achieved through the dual pH-sensitivity of PC, where it undergoes hydrolysis at pH 5.0, resulting in a change in surface charge from negative to positive, increasing interaction with negatively charged endosomal membranes. At pH 6.5, PC undergoes protonation, causing a change in surface charge from positive to negative, reducing interaction with negatively charged lysosomal membranes (173). Xia et al. developed a cancer vaccine based on porous silicon microparticles (PSM) loaded with growth factor receptor 2 (HER2) antigen. After being engulfed by dendritic cells, these particles exhibited prolonged early endosomal localization and enhanced cross-presentation. They induced type I interferon responses through TRIF and MAVS-dependent pathways, resulting in potent CD8^+^ T cell-dependent anti-tumor immunity in HER2-positive breast tumor-bearing mice (174).

Polymer/metal organic frameworks (MOFs) are a class of crystalline materials characterized by the coordination of metal ions with multidentate organic ligands. They possess high porosity and flexible functionality, making them ideal candidates for biomedical applications such as drug delivery and magnetic resonance imaging (175). Duan et al. engineered MOFs to load tumor-associated antigens. Due to the relatively unstable metal-ligand bonds, they can degrade in the acidic environment of endosomes/lysosomes, thereby releasing enhanced antigen cross-presentation. Additionally, MOFs introduce immunostimulatory CpG through Watson-Crick base pairing, further enhancing cytotoxic T lymphocyte responses in B16-OVA melanoma (176). In a study by Ma et al., a self-adjuvanted nano-vaccine was designed. This vaccine utilized a pH-sensitive galactosylated dextran-retinaldehyde (GDR) nanogel as a carrier and the patient’s own tumor cell lysate as an antigen, creating a personalized tumor immunotherapy strategy. The nanogel exhibits pH sensitivity, allowing it to release antigens and retinaldehyde within acidic lysosomes. The retinaldehyde activates the retinoic acid receptor (RAR) signaling pathway, promoting DCs maturation. Additionally, it induces lysosome rupture, leading to intracellular ROS generation, thereby enhancing proteasomal activity and MHC-I-mediated antigen cross-presentation, activating CTL responses, and suppressing the differentiation and function of Tregs and TAMs (95).

## 9 Clinical strategies for lysosome-based cancer therapy derived from fundamental research

Cancer vaccines are an immunotherapeutic approach that harnesses the immune system to effectively eliminate malignant cells. However, the current efficacy of cancer vaccines is suboptimal, primarily due to imprecise and uncontrollable antigen and adjuvant delivery and release (177).

The use of nucleic acids for cancer therapy. However, primary immune cells present inherent challenges in terms of efficiency and low activity compared to tumor cells. For targeting TAMs, recombinant bacterial ghosts have been developed to carry plasmid DNA and linear double-stranded DNA, localized within the lumen of the bacterial envelope rather than on the outer surface (178, 179). Loading ghosts with shLAMP2a allows for the stable modulation of peroxiredoxin 1 (PRDX1) and CREB-regulated transcription coactivator 1 (CRTC1) within TAMs, both of which serve as responsive factors activating the ROS signaling pathway, thereby inducing macrophage inflammation and controlling tumor growth (180). Unfortunately, strategies based on nucleic acid transfection have not yet been applied in clinical experiments and require extensive preliminary exploration. A summary of these preclinical experiments is provided in **Table 2**.

**Table 2:** Preclinical experiments targeting lysosomes.

Classification	Classification	Delivery system	Intervention	Effect
Cancer vaccine	Nucleic acid transfection	PEG = MT/PC NPs	VEGF siRNA, PIGF siRNA	NPs specifically targets M2-TAMs and breast cancer cell endocytosis and endosome/lysosome escape through double PH-sensitive charge conversion, inhibiting tumor growth and lung metastasis
		Ghost	shLAMP2A	Degradation of LAMP2a, PRDX1, and CRTC1 to promote pro-tumor activation of macrophages.
		CRISPR/Cas9	LAMP2A	Same as above
		lipid-coated calcium phosphate NP	PD-L1 siRNA and TRP2 mRNA	Promote the rapid internalization of lysosome region in lymph node dendritic cells and the downregulation of PD-L1, significantly promote T cell activation and proliferation.
		DNA nanomachine	TLR agonists dsRNA and CpG DNA	Antigen-presenting cells release antigens and adjuvants in lysosomes, activating TLR signaling pathways and antigen peptide presentation, inducing a strong antigen-specific cytotoxic T lymphocyte response.
	Antigen	Porous silicon micro-particles(PSM)	HER2	Psm-supported antigens exhibit extended early endosomal localization and enhanced cross-presentation via lysosomal dependent pathways, inducing dendritic cell type I interferon responses via TRIF and MAVS signaling.
		Metal-organic frameworks	OVA, CpG DNA	Enhances Th1 immune response.
Immunoadjuvant		Acetylated chondroitin sulfate-protoporphyrin	Imiquimod (R837)	R837 binds to the TLR-7 receptor on the lysosomal membrane of TAMs, stimulating the maturation of TAMs, thereby inducing an anti-tumor immune response.

TLRs primarily serve as sentinels for detecting and identifying various distinct molecular patterns associated with diseases, known as pathogen-associated molecular patterns (PAMPs). Upon TLR activation, they can induce signaling pathways dependent on either myeloid differentiation primary response 88 (MyD88) or TIR domain-containing adapter-inducing interferon-β (TRIF), ultimately leading to the activation of NF-κB, the secretion of cytokines and chemokines, and the initiation of both innate and adaptive immune responses (181). In the context of cancer, the presence of damage-associated molecular patterns (DAMPs) within tumor cells stimulates the activation of TLRs on immune cells within the TME, resulting in chronic inflammation. These alterations are interconnected with changes in the progression of tumors, inhibition of apoptosis, and the resistance of tumors to immune responses (182).

As a result, applying TLR agonists in cancer immunotherapy holds promise for transforming “cold” tumors into “hot” ones. This approach addresses the challenge of low response rates to single ICI and enhances the overall effectiveness of immunotherapy (177). Members of the TLR family, including TLR3, TLR7, TLR9, and TLR10, are situated within the endosomal/lysosomal compartment, and their ligands encompass double-stranded RNA (dsRNA), single-stranded RNA (ssRNA), CpG DNA, and others. Ligands for TLR agonists employ the innate immune pathways to modulate the type of immune response generated by vaccines (183).

Currently, several clinical trials are either underway or have been completed involving TLR agonists, mTOR inhibitors, and Hydroxychloroquine (lysosome inhibitors) in combination with cancer immunotherapy, as outlined in **Table 3**. For instance, Xiao et al. have developed polymer micelles designed to target TLR7. These micelles enable the specific delivery of R837 to TAMs, prompting their maturation. This, in turn, triggers anti-tumor immune responses and mitigates immune suppression within the TME (184).

**Table 3:** Interventions targeting lysosomes.

Drug	Disease	Intervention	Stage of development	NCT number	Progress situation
Poly-ICLC (TLR3 agonist)	Hepatocellular Carcinoma	Nivolumab+ poly-ICLC	Phase I	NCT05281926	Recruiting
	Colon cancer	Pembrolizumab + Poly-ICLC (TLR3 agonist)	Phase I/II	NCT02834052	Completed
	Nonspecific cancer	Poly-ICLC + Nivolumab/Pembrolizumab + Atezolizumab/Durvalumab	Phase I/II	NCT03721679	Terminated
	NSCLC	Poly-ICLC + IVIG	Phase I/II	NCT06064279	Not yet recruiting
	Glioblastoma	IMA950/Poly-ICLC +pembrolizumab	Phase I/II	NCT03665545	Active, not recruiting
	Pancreatic Adenocarcinoma	Poly-ICLC + Dendritic Cells	Phase I	NCT01677962	Completed
	Myelodysplastic Syndrome or Acute Myeloid Leukemia	DEC-205/NY-ESO-1 Fusion Protein CDX-1401, Poly ICLC, Decitabine, and Nivolumab	Phase I	NCT03358719	Completed
Rintatolimod (TLR3 agonist)	Ovarian cancer	Cisplatin + Pembrolizumab + Rintatolimod (TLR3 agonist)	Phase I/II	NCT03734692	Recruiting
	Pancreatic Ductal Adenocarcinoma	Durvalumab + Rintatolimod	Phase I/II	NCT05927142	Not yet recruiting
	Triple Negative Breast Cancer	Rintatolimod + Celecoxib +Interferon Alfa-2b + Pembrolizumab	Phase I/II	NCT05756166	Not yet recruiting
	Peritoneal Surface Malignancies	αDC1 Vaccine + Celecoxib+Interferon Alfa-2b + Rintatolimod	Phase I/II	NCT02151448	Completed
	Ovarian Cancer	Rintatolimod + Pembrolizumab + Cisplatin	Phase I/II	NCT0373469	Recruiting
	Breast Cancer	HER-2/neu peptide vaccine + sargramostim + Rintatolimod	Phase I/II	NCT01355393	Completed
	Colorectal Cancer Metastatic to the Liver	Celecoxib + Recombinant Interferon Alfa-2b+Rintatolimod	Phase II	NCT03403634	Completed
Ampligen (TLR3 agonist)	Pancreatic Ductal Adenocarcinoma	Durvalumab + Ampligen	Phase I/II	NCT05927142	Not yet recruiting
BNT411 (TLR7 agonist)	Small cell lung cancer	Etoposide + Carboplatin + Atezolizumab + BNT411 (TLR7 agonist)	Phase I/II	NCT04101357	Active, not recruiting
Imiquimod (TLR7 agonist)	Melanoma	Pembrolizumab + Imiquimod	Phase I	NCT03276832	Active, not recruiting
	Malignant Glioma	Dendritic Cell Vaccine+ Imiquimod	Phase I	NCT01792505	Completed
	Ovarian Cancer	Dendritic Cell Vaccine + GM-CSF + Imiquimod	Phase II	NCT00799110	Active, not recruiting
	Ependymomas	HLA-A2 restricted synthetic tumor antigen + Imiquimod	Phase I	NCT01795313	Recruiting
SHR2150 (TLR7 agonist)	Nonspecific cancer	SHR2150+anti-PD-1 antibody and/or anti-CD47 antibody	Phase I/II	NCT04588324	Unknown
Resiquimod (TLR7/8 agonist)	Melanoma	Resiquimod + Vaccine Therapy	Phase I	NCT00470379	Completed
	Nonspecific cancer	CDX-1401and Resiquimod and/or Poly-ICLC	Phase I/II	NCT00948961	Completed
	Melanoma	Gp100+Resiquimod+MAGE-3	Phase II	NCT00960752	Completed
MEDI9197 (TLR7/8 agonist)	Nonspecific cancer	Durvalumab + MEDI9197 (TLR7/8 agonist)	Phase I	NCT02556463	Terminated
CpG-7909 (TLR9 agonist)	Renal Cell Cancer	CpG-7909	Phase I/II	NCT00043407	Completed
	Prostate Cancer	NY-ESO-1 protein/CpG 7909	Phase I	NCT00292045	Completed
	Nonspecific cancer	NY-ESO-1 protein/CpG 7909	Phase I	NCT00299728	Completed
SD-101 (TLR9 agonist)	Pancreatic cancer	Radiation Therapy + Nivolumab + SD-101 (TLR9 agonist)	Phase I	NCT04050085	Completed
		Pebrolizumab+SD-101	Phase I	NCT05607953	Recruiting
	Liver Tumors	Pebrolizumab+SD-101+Ipilimumab	Phase I/II	NCT05220722	Recruiting
	Uveal Melanoma	Nivolumab+SD-101+Ipilimumab+Relatlimab	Phase I	NCT04935229	Recruiting
	Prostate Cancer	Pebrolizumab+SD-101+ILeuprolide acetate	Phase II	NCT03007732	Active, not recruiting
Tilsotolimod (TLR9 agonist)	Nonspecific cancer	Ipilimumab + Nivolumab + Tilsotolimod	Phase I	NCT04270864	Active, not recruiting
		Ipilimumab + Nivolumab + Tilsotolimod	Phase II	NCT03865082	Active, not recruiting
	Melanoma	Ipilimumab + Tilsotolimod	Phase II	NCT02644967	Completed
CMP-001 (TLR9 agonist)	Head and Neck Squamous Cell Carcinoma	Pembrolizumab+CMP-001	Phase II	NCT04633278	Active, not recruiting
	Melanoma	Nivolumab+CMP-001	Phase III/III	NCT04695977	Active, not recruiting
	Non-Small Cell Lung Cancer	Atezolizumab+CMP-001	Phase I	NCT03438318	Completed
	Prostate Cancer	Nivolumab + VLP-encapsulated CMP-001	Phase II	NCT05445609	Recruiting
	Metastatic Colorectal Cancer	Nivolumab + Liver radiation therapy+ Ipilimumab+CMP-001	Phase I	NCT03507699	Completed
Hydroxychloroquine (Lysosome Inhibitor)	Gastrointestinal cancer	Cobimetinib + Atezolizumab + Hydroxychloroquine	Phase I/II	NCT04214418	Active, not recruiting
	Melanoma	Nivolumab + Hydroxychloroquine + Ipilimumab	Phase I/I	NCT04464759	Recruiting
	Malignancies	Cobimetinib + Hydroxychloroquine + Atezolizumab	Phase I/II	NCT04214418	Active, not recruiting
	Breast Cancer	HCQ + Avelumab/Hydroxychloroquine + Palbociclib	Phase II	NCT04841148	Recruiting
	Pancreatic Ductal adenocarcinoma	Nivolumab/HydroxychloroquineIpilimumab + nP/gem	Phase I	NCT04787991	Active, not recruiting
Rapamycin (mTOR Inhibitor)	Nonspecific cancer	Nab-Rapamycin + Nivolumab	Phase I/II	NCT03190174	Completed
Temsirolimus (mTOR Inhibitor)	Nonspecific cancer	Temsirolimus + Irinotecan + capecitabine + nivolumab	Phase I/II	NCT02423954	Terminated
	Kidney Cancer	Recombinant interferon alfa + temsirolimus	Phase I	NCT00045370	Completed
	Multiple Myeloma	RAPA-201 Autologous T cells	Phase II	NCT04176380	Unknown
		Rapamycin-Generated Autologous Th1/Tc1 Cells (modified primary human T cells)	Phase I/II	NCT01239368	Terminated
GNS651 (Autophagy inhibitor)	Nonspecific cancer and SARS-CoV-2 Infection	GNS651+Avdoralimab+Monalizumab	Phase II	NCT04333914	Completed

+: combination; /: or.

Furthermore, Ding et al. have designed and produced a cancer vaccine based on DNA nanomachines. This vaccine is created through the precise assembly of two types of molecular adjuvants (TLR agonists dsRNA and CpG DNA) and antigen peptides within the interior of tubular DNA nanostructures. The DNA nanomachine can be activated by the low pH within lysosomes of APCs, leading to the unfolding of its structure and the release of antigens and adjuvants. This activation subsequently triggers the TLR signaling pathways and the presentation of antigen peptides, resulting in a potent antigen-specific CTL response. The vaccine induces noticeable maturation of APCs, antigen-specific CTL responses, and subsequently, tumor regression. Additionally, the vaccine establishes long-lasting T-cell memory responses that effectively safeguard mice against tumor rechallenge (185).

Adoptive T-cell therapy, including chimeric antigen receptor T-cell therapy, TILs therapy, and endogenous T-cell therapy, involves the isolation of T-cells from a patient’s peripheral blood.

These T-cells are then activated and enhanced for tumor specificity through biological or genetic engineering techniques before being expanded and reintroduced into the peripheral blood. While adoptive T-cell immunotherapy has been approved for the treatment of B-cell lymphomas, its success rates in solid tumors are comparatively lower.

CAR is an artificially designed molecule that enables T-cells to recognize and eliminate tumor cells expressing specific antigens.

CAR-T cell therapy is an immunotherapeutic approach that utilizes genetic engineering to introduce CARs into a patient’s own T-cells. It has demonstrated significant efficacy in certain types of cancer (186). However, CAR-T cell therapy still faces limitations regarding its persistence and functionality within the body, resulting in tumor relapse or resistance in some patients (187).

Lysosomes play a negative regulatory role in CAR-T cells by degrading CAR, thereby reducing CAR-T cell activity and persistence. Research by Wang et al. revealed that after binding with tumor antigens, CAR undergoes ubiquitination and subsequent internalization, leading to degradation in lysosomes, which diminishes CAR-T cell activity and persistence. To counteract this process, they designed a modified CAR (rCAR) by mutating all lysine residues in the cytoplasmic region of CAR, making it less susceptible to ubiquitination and downregulation. Compared to traditional CARs, rCAR can reappear on the cell surface after internalization and continue to function within intracellular compartments. In mouse tumor models, rCAR-T cells exhibited higher surface CAR levels, enhanced cytotoxicity, prolonged survival, and improved anti-tumor effects (188). This innovative rCAR design holds promise for enhancing the safety and effectiveness of clinical CAR-T cell therapy.

Perforin and granzymes are two essential weaponry components required for T-cell-mediated killing of tumor cells or infected cells (189, 190).

Lysosomes are responsible for storing and transporting perforin and granzymes. When T cells form an immune synapse with target cells, lysosomes release perforin and granzymes at the synapse. Perforin can form holes in the cell membrane of the target cells, and granzyme can enter the cytoplasm of the target cells through these holes and trigger apoptosis of the target cells (189, 191). Adoptive T-cell therapy faces a challenge in solid tumors due to their low responsiveness, possibly due to suppressed T-cells displaying weak tumor targeting and reduced activation in the immunosuppressive TME, leading to a sharp reduction in the delivery of potent toxins to the tumor region. Therefore, harnessing the synthetic and release capabilities of lysosomes is crucial to enhance the effectiveness of T-cell immunotherapy. Zhang et al. have developed an adoptive T-cell vehicle (ATV) loaded with lysosome-reactive nanoparticles (LYS-NP). LYS-NP consists of a MOF as a substrate structure and a biotin-modified lysosome-targeting aptamer (CD63-aptamer). Perforin and granzyme B can be loaded onto the MOF, which is then engulfed by T-cells and localized within lysosomes. In an acidic environment, the MOF degrades, releasing perforin, granzyme B, and Ca^2+^, leading to their substantial accumulation within lysosomes. When the T-cell receptor (TCR) binds with MHC to form an immune synapse, lysosomes autonomously release their contents, thereby enhancing the killing capability of T cells (192). This novel strategy of reprogramming T-cell lysosomes to augment their anti-tumor effect provides a new, efficient, safe, controllable, and adjustable approach for solid tumor immunotherapy, offering significant clinical application potential.

## 10 Conclusion

This article provides an overview of the physiological functions of lysosomes, their role in the TME, and potential tumor immunotherapy approaches targeting lysosomes. Lysosomes serve as not only the cellular degradation centers but also play crucial roles in immune regulation, nutrient sensing, and cellular communication processes. Recent research has shown that lysosomal homeostasis has a dual role in tumor initiation and progression. It can activate immune surveillance by processing and presenting tumor antigens and regulating inflammation, while also supporting cancer cell metabolism and resistance to stress, facilitating tumor progression.

Current evidence highlights the critical importance of lysosomes in disease. A deeper understanding of lysosome-related mechanisms will enhance our ability to leverage their biological properties, thereby increasing the efficacy of tumor immunotherapy across various stages of immune response. During the antigen presentation stage, lysosomes are responsible for antigen processing within APCs, binding to MHC-II, and presenting them on the cell membrane. In tumor cells, the use of lysosomes or autophagy inhibitors can restore the membrane expression of MHC-I. In the T-cell activation process, lysosomes can enhance the degradation or transport of immune checkpoint molecules like PD-1, PD-L1, and CTLA4, reducing immune evasion by tumor cells. In the recruitment and infiltration of immune cells, lysosomes participate in the release of various adhesion factors, chemokines, and the regulation of selectins. Additionally, lysosomes can promote tumor nutrient adaptation and immune suppression by modulating cellular autophagy and metabolism.

In tumor therapy, various approaches targeting lysosomes have shown promising results. When combined with immunotherapy, these methods may further improve treatment outcomes. Examples include using lysosomal acidic environments for pH-sensitive nanoparticle drug delivery and release, avoiding lysosomal degradation of CAR in ACT therapy, and using lysosome or autophagy inhibitors, TLR agonists in combination with DCs vaccines or ICIs, among others. It’s important to note that the role of autophagy in tumor progression varies at different stages and in different cell types. Therefore, interventions to inhibit or stimulate autophagy should be carefully chosen and implemented.

In conclusion, while targeting lysosomes or harnessing their properties represents a potential effective strategy for tumor immunotherapy, the mechanisms and methods related to lysosomes in tumor immunotherapy are not fully understood at present. Further basic and clinical research is needed to address issues related to lysosomal drug delivery, specificity, resistance, and personalized treatment plans, with the aim of providing better treatment options for cancer patients.

## Author contributions

YX: Writing – original draft. BS: Writing – original draft. YZ: Writing – review & editing.
